# Exploring biomechanical variations in ankle joint injuries among Latin dancers with different stance patterns: utilizing OpenSim musculoskeletal models

**DOI:** 10.3389/fbioe.2024.1359337

**Published:** 2024-04-10

**Authors:** Xiangli Gao, Datao Xu, Julien S. Baker, Teo Ee-Chon, Minjun Liang, Yaodong Gu

**Affiliations:** ^1^ Faculty of Sports Science, Ningbo University, Ningbo, China; ^2^ Faculty of Engineering, University of Pannonia, Veszprem, Hungary; ^3^ School of Mechanical and Aerospace Engineering, Nanyang Technological University, Singapore, Singapore; ^4^ Department of Radiology, Ningbo No. 2 Hospital, Ningbo, China

**Keywords:** Latin dancers, ankle sprain, muscle force, biomechanics, stance patterns

## Abstract

**Background:** Dancers represent the primary demographic affected by ankle joint injuries. In certain movements, some Latin dancers prefer landing on the Forefoot (FT), while others prefer landing on the Entire foot (ET). Different stance patterns can have varying impacts on dancers’ risk of ankle joint injuries. The purpose of this study is to investigate the differences in lower limb biomechanics between Forefoot (FT) dancers and Entire foot (ET) dancers.

**Method:** A group of 21 FT dancers (mean age 23.50 (S.D. 1.12) years) was compared to a group of 21 ET dancers (mean age 23.33 (S.D. 0.94) years), performing the kicking movements of the Jive in response to the corresponding music. We import data collected from Vicon and force plates into OpenSim to establish musculoskeletal models for computing kinematics, dynamics, muscle forces, and muscle co-activation.

**Result:** In the sagittal plane: ankle angle (0%–100%, *p* < 0.001), In the coronal plane: ankle angle (0%–9.83%, *p* = 0.001) (44.34%–79.52%, *p* = 0.003), (88.56%–100%, *p* = 0.037), ankle velocity (3.73%–11.65%, *p* = 0.017) (94.72–100%, *p* = 0.031); SPM analysis revealed that FT dancers exhibited significantly smaller muscle force than ET dancers around the ankle joint during the stance phase. Furthermore, FT dancers displayed reduced co-activation compared to ET dancers around the ankle joint during the descending phase, while demonstrating higher co-activation around the knee joint than ET dancers.

**Conclusion:** This study biomechanically demonstrates that in various stance patterns within Latin dance, a reduction in lower limb stance area leads to weakened muscle strength and reduced co-activation around the ankle joint, and results in increased ankle inversion angles and velocities, thereby heightening the risk of ankle sprains. Nevertheless, the increased co-activation around the knee joint in FT dancers may be a compensatory response for reducing the lower limb stance area in order to maintain stability.

## 1 Introduction

The ankle joint, located at the convergence of the lower leg and the foot (Palastanga et al., 2006), assumes a critical role in providing structural stability and facilitating biomechanical support for the body (Simon et al., 2014). It facilitates a diverse spectrum of movements, encompassing flexion, extension, rotation, and lateral shifts in the foot (Palastanga et al., 2006; Khuyagbaatar et al., 2024). Consequently, once an individual’s ankle joint is injured, it not only limits physical activities but also hinders daily life (Houston et al., 2015; Hubbard-Turner and Turner, 2015; Wright et al., 2017). This joint is particularly susceptible to injuries during athletic activities (Taghavi Asl et al., 2022), predominantly encompassing ankle sprains, ligament strains, muscle tears, among others. Among these injuries, ankle sprains are a particularly common form of injury (Guillo et al., 2013), accounting for approximately 15% of all injuries (Garrick, 1977; Fong et al., 2007; Hootman et al., 2007; Doherty et al., 2014). For example, in the context of basketball, athletes frequently engage in high-intensity maneuvers such as jumping and rapid directional changes, presenting a significant challenge to the stability of basketball players (Hertel, 2002). Additionally, athletes often collide or come into contact with opponents, further impacted by the inherent physicality of the performance. A robust biomechanical foundation is required to effectively manage the weight and force encountered during these physical engagements. When the ankle joint’s ability to maintain balance significantly decreases, susceptibility to ankle sprains notably increases (McKay et al., 1996; Messina et al., 1999; Kovács et al., 2023).

Ankle sprain issues are also prevalent in the field of dance (Russell, 2010), with reports indicating that approximately 90% of dancers experience injuries over the course of their extensive dance careers, and the ankle and foot account for approximately 40% of all injuries (Garrick, 1977; Schafle et al., 1990). In terms of injury types, among every 100 contemporary dancers, the proportion of foot and ankle injuries ranges from 17% to 24% (Garrick, 1977; Nilsson et al., 2001; Byhring and Bø, 2002; Bronner et al., 2003; Kadel, 2006; Simon et al., 2014). In ballet, the incidence rate of foot injuries ranges from 65% to 79% (O'Loughlin et al., 2008; Costa et al., 2016; Hung et al., 2021). As Latin dance gains in popularity, the community of Latin dance enthusiasts continues to grow, making the provision of scientific guidance increasingly crucial. It is worth noting that compared to ballet, there has been relatively less research on lower limb biomechanics in Latin dance. Nevertheless, in recent years, more and more researchers have shifted their focus towards Latin dance. Some studies suggest that Latin dance can enhance body balance (Kiliç and Nalbant, 2022; Liu et al., 2022), further highlighting its potential for rehabilitative interventions in individuals with Parkinson’s disease (Hulbert et al., 2017; Ismail et al., 2021). Despite its benefits for balance improvement, the intricate movements involved in Latin dance also present a notable risk of ankle injuries. Dancers need to engage in extensive ankle flexion, extension, turning, and rotation during dance training, which may lead to overstretching or twisting of the ankle muscles. Research indicates that the probability of lower limb injuries in sports dance is 34.3%, with the likelihood of ankle joint injuries at 23.5% and knee joint injuries at 15.7% (Kuisis et al., 2012). It is evident that there is a relatively high probability of ankle joint injuries. Although previous research has focused on the injury concerns of Latin dancers, we have found that there is relatively limited research on lower limb injuries among dancers. The lower limbs play a crucial role in dance, and any injury to them can have a detrimental impact on a dancer’s career. Therefore, in-depth research into the biomechanical characteristics of dancers’ lower limbs has become particularly important. In the flawless rendition of Latin dance, seamless coordination between male and female dancers is imperative. In Latin dance, males primarily take on the roles of leading and partnering, while females, akin to the core within a flower, play a crucial role. Diverging from their male counterparts, female dancers showcase their footwork in a more intricately woven manner, encompassing various spins, precisely delineated rhythmic divisions, and elegant movements involving ankle flexion and extension. The complexity of these movements demands proficient posture control abilities (Leanderson et al., 1996) as the foundation for maintaining balance, and prolonged practice induces adaptive changes in biomechanics (Ödemiş et al., 2022), particularly in relation to the ankle joints.

In specific Latin dance movements, dancers adopt diverse body gravity distribution patterns, some favoring the FT while others distributing it across the ET. In the kicking movement of Jive, the process primarily consists of three stages: draw or lift (where the dancer lifts the leg, bringing the leg curve towards the body, preparing for the next move), extend or kick (where the dancer quickly stretches the leg from the drawn position to complete the kicking action, reaching the highest point of the leg movement), and return or drop (where the dancer, after the kicking motion, swiftly returns the leg to the initial position or prepares for the subsequent dance move). The combination and fluid transition of these stages are crucial in the Jive kicking action. When executing the kicking motion, FT dancers have their supporting leg with the half foot on the ground, and the body center consistently placed over the front ball of the foot. In contrast, ET dancers, during the kicking motion, maintain the supporting leg with the entire foot on the ground, and the body center is positioned over the dancer’s entire foot. These distinct leg support methods and body center positions result in noticeable differences in the kicking movements between the two types of dancers. Previous research has indicated that alterations in the lower limb stance area might lead to changes in the musculoskeletal structure of the lower limbs (Polat and Kabakcı, 2021). FT dancers typically have a smaller lower limb stance area compared to ET dancers. Consequently, FT dancers may adapt their lower limb usage during dancing to accommodate the reduced stance area. In the evaluation of the significance of foot functionality and the preservation of body posture stability, the pivotal roles played by muscle activation and muscle synergy come to the forefront (Cai et al., 2023). Additionally, the FT movement pattern increases plantar flexion at the ankle joint, effectively increasing the distance between the heel and the ground, akin to an increase in heel height. Studies have confirmed a correlation between heel height and an increased risk of foot injuries (Polat and Kabakcı, 2021). Moreover, the use of high-heeled shoes may increase the risk of ankle sprains (Ebbeling et al., 1994; Gajdosik et al., 1999). Based on this research, we speculate that FT dancers might be more prone to ankle joint sprains compared to ET dancers.

Therefore, the main objective of this study is to systematically explore the variation in ankle joint sprain risk among Latin dancers under different support modes. Through in-depth analysis and comparison of dancers’ ankle joint kinematics, dynamics, and lower limb muscle activity, we aim to thoroughly investigate the impact of these support modes on the biomechanics of the dancers’ lower limbs from a biomechanical perspective. Ultimately, we aspire to offer more precise insights through scientific research to assist dancers in effectively mitigating potential injury risks, thereby extending their sustainable development in the field of dance profession.

## 2 Materials and methods

### 2.1 Participants

Based on previous research, we calculated the sample size determination using G- Power software (version: 3.1.9.7; Henry University of Düsseldorf, Düsseldorf, Germany). An independent samples t-test was conducted, with an effect size of 0.8 (significance level: 0.05) (Gao et al., 2023). In this experiment, a total of 21 dancers habitually placing their body weight on the forefoot while dancing (age: 23.50 ± 1.12 years; height: 165.50 ± 2.92 cm; body weight (BW): 53.13 ± 2.52 kg), and the remaining 21 habitually distributing their body weight across the entire foot (age: 23.33 ± 0.94 years; height: 165.89 ± 2.64 cm; body weight (BW): 52.22 ± 2.48 kg) were investigated. The participants all had at least 5 years of dance experience, with a minimum of two or more professional training sessions per week. All participants were free from any injuries for the past 6 months prior to data collection. All were informed about the study procedures, conditions, and requirements, and provided written informed consent before data collection. This study was approved by the Ethics Review Committee of Ningbo University (Approval Code: RAGH20230620).

### 2.2 Experimental procedure

This experiment was conducted in the Sports Biomechanics Laboratory at Ningbo University. Drawing from prior research, we affixed 38 standard markers, each with a diameter of 12.5 mm, onto the participants to precisely capture their motion trajectories (Zhou et al., 2021). Eight infrared cameras were utilized to record motion, and the Vicon motion capture system was intricately combined with a force plate (AMTI, Watertown, MA, United States) for the comprehensive acquisition of both kinematic and kinetic data. The sampling frequencies for kinematics and kinetics were 200 and 1,000 Hz (Xu et al., 2022), respectively. The EMG system (Delsys, Boston, Massachusetts, United States) was used to collect surface muscle activation and force data at a frequency of 1,000 Hz (Cai et al., 2023; Cai et al., 2024). Surface electromyography (EMG) sensors were placed on the subjects’ vastus medialis, vastus lateralis, rectus femoris, tibialis anterior, medial gastrocnemius, and lateral gastrocnemius muscles. Maximum Voluntary Contractions (MVC) were also collected for these six muscle groups to standardize muscle activation.

**FIGURE 1:**
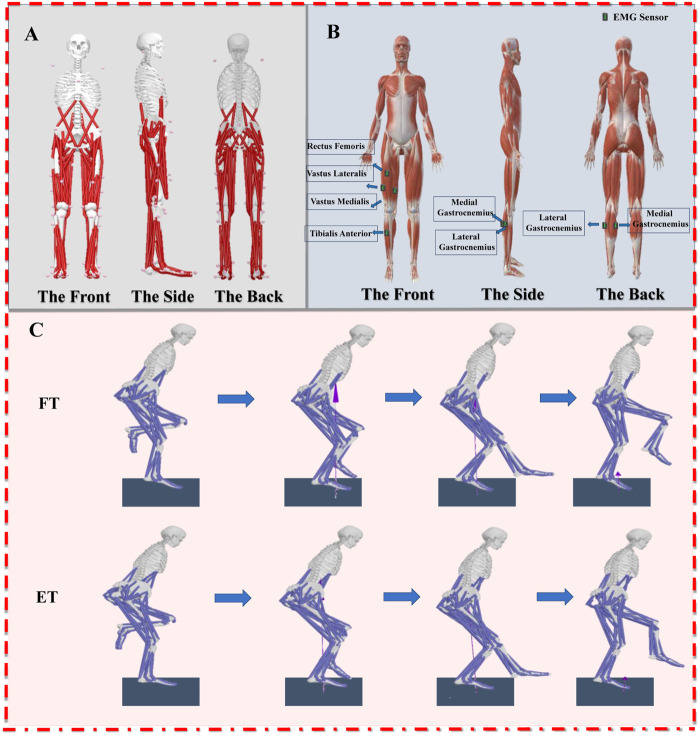
**(A)**: Illustration of the musculoskeletal model; **(B)**: Illustration of the EMG acquisition; **(C)**: Illustration of the motion capture process (FT: Forefoot; ET: Entire foot).

Prior to the formal experiment, participants were instructed to wear specialized Latin dance shoes with 7.5 cm heels and attire featuring a snug fit, facilitating warm-up procedures. Subsequently, the participants were familiarized with experimental environments and the experimental procedures. In the formal experiment, participants were required to provide a set of static data (Xu et al., 2023). They received instructions to stand in an anatomical position, step onto the force plate, upon hearing a command, and prepare for data collection. The data was collected as participants followed the rhythm of the music, performing kicking movements in the jive from one end of the force platform to the other. Throughout the entirety of the experiment, the dancers maintained a consistent posture, with both hands gracefully resting on their waists. Participants were specifically instructed to coordinate the contact of their right foot with the force plate on the “two” beat (as shows in the Figure 1). The data collection commenced when the ground reaction force exceeded 10 N (Xu et al., 2024). When collecting surface electromyography, it was necessary to remove excess hair from the test areas to reduce impedance at the skin-electrode interface (Xu et al., 2023). Throughout the experiment, researchers closely monitored participant performance. In instances where a participant deviated from the music rhythm or failed to fully place their foot on the force plate, the trial was deemed invalid, prompting the repetition of measurements to ensure accuracy and reliability of the data.

### 2.3 Data processing and analysis

The Vicon Nexus software was employed to export data in c3d format for the acquisition of participants’ kinematic and kinetic data (Li et al., 2022). Subsequently, the data undergoes processing using MATLAB R2022a (The MathWorks, Natick, MA, United States) (Cai et al., 2023), involving operations such as coordinate transformation, low-pass filtering, data extraction, and format conversion. The coordinate systems of kinematic and kinetic data were transformed into the coordinate system used in subsequent simulations. Biomechanical data pertaining to kinematics and ground reaction forces were subjected to filtering using fourth-order zero-phase-lag Butterworth low-pass filters with cutoff frequencies set at 10 and 20 Hz. Kinematic and ground reaction force data were extracted and transformed into the trc format (marker trajectories) and force plate data format essential for the OpenSim simulation software. OpenSim (Stanford University, Stanford, CA, United States) was employed in this study for the processing and computation of biomechanical parameters. Static models were imported into OpenSim 4.4 software, and the scale tool was utilized to obtain body measurement models for each participant. Muscle origin and insertion points were identified to align with the limb lengths of the participants. Using the inverse kinematics (IK) tool in OpenSim 4.4 software, joint angles during the stance phase of kicking movements were computed, and motion files (mot) were created. The residual reduction algorithm (RRA) was applied to smooth the kinematic data, improving the accuracy of dynamic data and making it consistent with the measured data. The calculation of Center of Mass (CoM) position and velocity was achieved through consecutive utilization of OpenSim’s Inverse Kinematics and Body Kinematics Tools.

**FIGURE 2:**
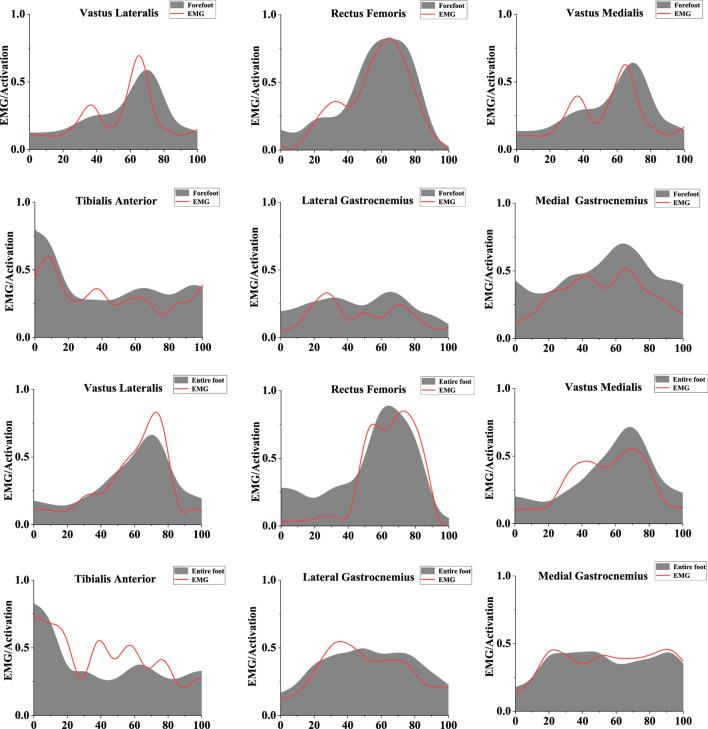
Illustration of the EMG/activation of muscle. The red line represents the results of EMGc activation, and the gray shaded area represents musculoskeletal modeling activation results. The left scale ranges from 0 to 1, indicating muscle activation from no activation to full activation. The bottom scale ranges from 0 to 100, representing the stance phase.

Muscle activation and muscle forces were determined using static optimization, employing the smoothed kinematic data obtained during the process. The EMG data underwent a fourth-order band-pass filtering from 10 to 500 Hz to prepare for full-wave rectification. Subsequently, a 10 Hz low-pass filter was applied to further refine and smooth the data (Zhou et al., 2021). At the same time, the EMGc signals were normalized by dividing the EMG amplitude by the maximum root mean square amplitude, which was further divided by MVC to obtain the activation level of each muscle (Xu et al., 2023). EMGc activation variables were qualitatively compared with OpenSim simulated muscle activation to assess the reliability of the OpenSim model. The comparative findings, depicted in Figure 2, demonstrate a favorable correlation between the expected muscle activation throughout the stance phase and the electromyographic (EMGc) signals.

To derive the co-activation of lower limb muscles during the descending phase, based on prior research we employed the following formula (Márquez et al., 2013; Lin et al., 2019):

$$Muscle\;co-activation\;\left(\%\right)=\left(RMS\;EMG\;antagonist\;/\;RMS\;EMG\;agonist\right)\times 100$$

### 2.4 Statistical analysis

Prior to engaging in statistical analysis, the dataset was subjected to a Shapiro-Wilk normality test to evaluate the adherence of the data to a normal distribution. Subsequently, an independent *t*-test was employed to scrutinize distinctions between the two modes of movement. In the context of statistical parametric mapping (SPM) analysis, the entire dataset was extracted, and a bespoke MATLAB script was utilized to unfold the data from the stance phase into time-series curves comprising 101 data points. Following this data preparation, statistical analysis was carried out using the open-source SPM1d paired-sample *t*-test script, with a predetermined significance threshold established at *p* < 0.05.

## 3 Results

**FIGURE 3:**
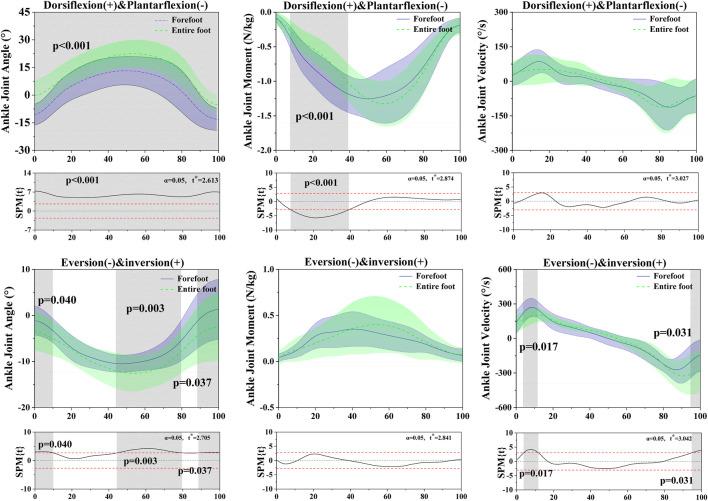
Illustration of the results between FT and ET the lower limb showing the statistical parametric mapping outputs for the ankle angle, moment, velocity during the stance phase. The values of t* are shown on the left of each image. Grey shades represent the significant differences and t-values of the SPM or all participants, dashed red lines represent the results at *p* = 0.05.

**FIGURE 4:**
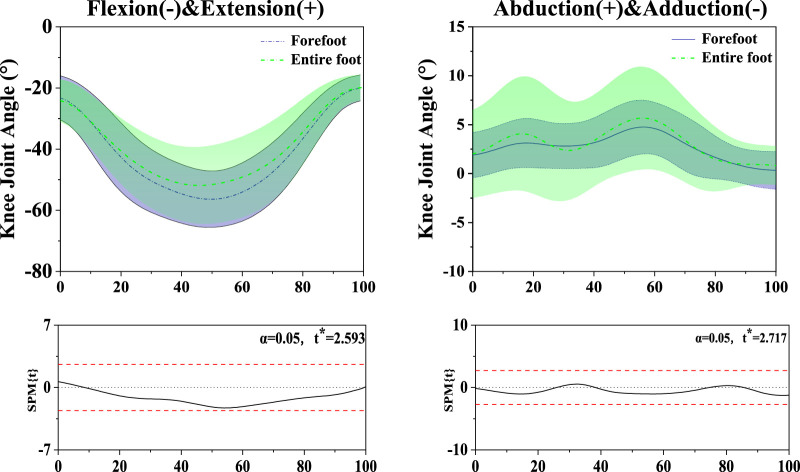
Illustration of the results between FT and ET the lower limb showing the statistical parametric mapping outputs for the knee angle during the stance phase. The values of t* are shown on the left of each image. Grey shades represent the significant differences and t-values of the SPM or all participants, dashed red lines represent the results at *p* = 0.05.

**FIGURE 5:**
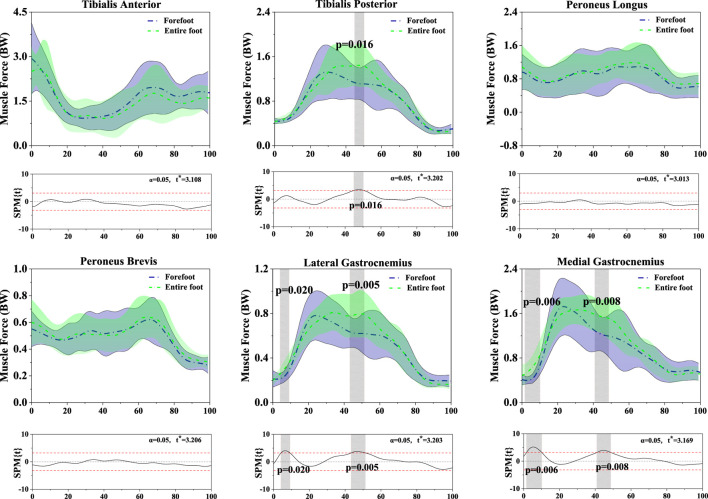
Illustration of the results between FT and ET the lower limb showing the statistical parametric mapping outputs for the Tibialis Anterior, Tibialis Posterior, Peroneus Longus, Peroneus Brevis, Lateral Gastrocnemius, and Medial Gastrocnemius during the stance phase. The values of t* are shown on the left of each image. Grey shades represent the significant differences and t-values of the SPM or all participants, dashed red lines represent the results at *p* = 0.05. BW: Body Weight.

**FIGURE 6:**
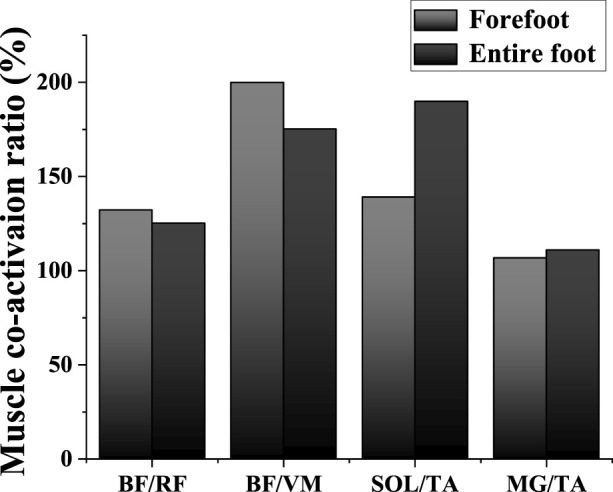
Illustration of lower limb muscle co-activation results between FT and ET during the descending phase. Abbreviations: TA: tibialis anterior; MG: medial gastrocnemius; BF: biceps femoris; RF: rectus femoris; VM: vastus medialis; SOL: soleus.

**FIGURE 7:**
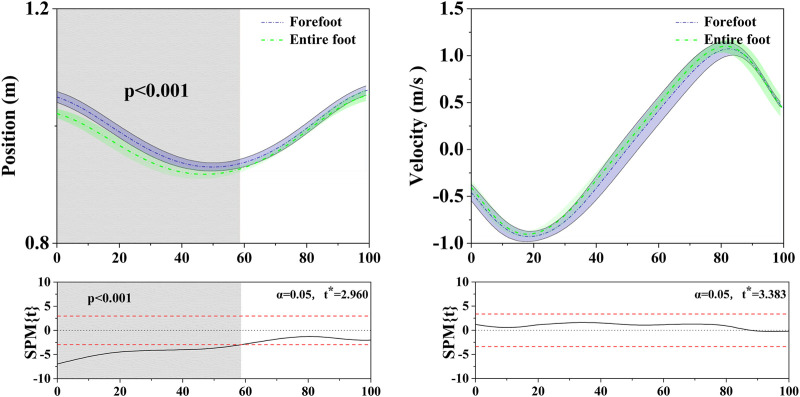
Illustration of COM vertical position and velocity results between FT and ET during the stance phase. The values of t* are shown on the left of each image. Grey shades represent the significant differences and t-values of the SPM or all participants, dashed red lines represent the results at *p* = 0.05.

**FIGURE 8:**
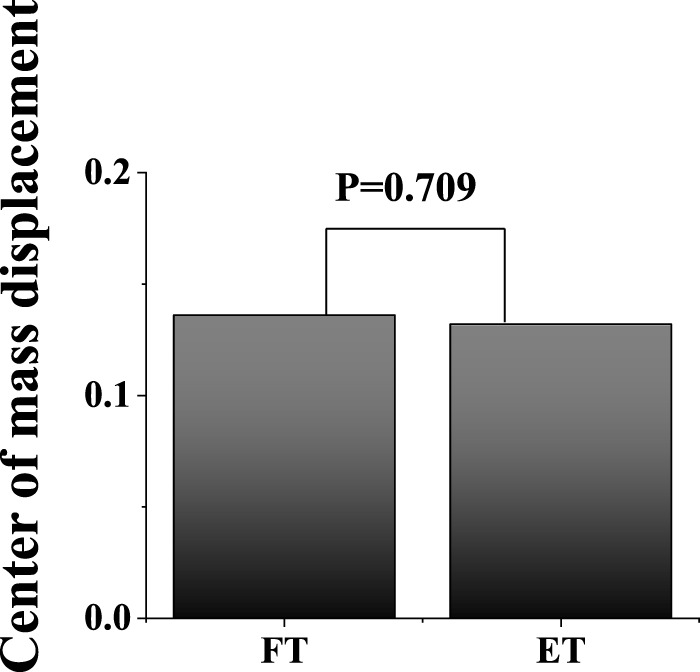
Illustration of COM displacement results between FT and ET during the stance phase.

Differences were found between FT dancers and ET dancers. Figure 3 shows the difference in ankle joint angle, joint moment and joint velocity between FT and ET during the stance phase. Figure 4 shows the difference in knee joint angle between FT and ET during the stance phase. Figure 5 shows the difference in Tibialis Anterior, Tibialis Posterior, Peroneus Longus, Peroneus Brevis, Lateral Gastrocnemius, and Medial Gastrocnemius between FT and ET during the stance phase. Figure 6 shows the difference in muscle co-activation ratio between FT and ET during descending phase. Figures 7, 8 shows the difference in COM between FT and ET during the stance phase.

### 3.1 Ankle angle, moment, velocity

The SPM analysis revealed the results of ankle joint kinematics and kinetics during the stance phase, comparing FT dancers with ET dancers. In the sagittal plane: ankle angle (0%–100%, *p* < 0.001), ankle moment (7.67%–39.17%, *p* < 0.001), SPM analysis revealed in the ankle velocity that there was no significant difference between FT and ET during the stance phase. In the coronal plane: ankle angle (0%–9.83%, *p* = 0.001) (44.34%–79.52%, *p* = 0.003), (88.56%–100%, *p* = 0.037), ankle velocity (3.73%–11.65%, *p* = 0.017) (94.72–100%, *p* = 0.031). SPM analysis revealed in the ankle moment that there was no significant difference between FT and ET during the stance phase. Table 1 displays significant differences in ankle dorsiflexion angle (*p* < 0.001), ankle plantarflexion angle (*p* < 0.001), ankle version angle (*p* < 0.001), ankle inversion velocity (*p* < 0.001).

**TABLE 1:** Comparison of all joint angle, moment, velocity variables change between FT dancers and ET dancers during the stance phase.

	Parameters	Peak value	FT mean ± SD	ET mean ± SD	*p*-value
Sagittal plane	Angle (◦)	Dorsiflexion	14.33 ± (8.12)	22.90 ± (7.47)	<0.001*
		Plantarflexion	−15.06 ± (4.53)	−7.57 ± (5.55)	<0.001*
	Moment (Nm/kg)	Dorsiflexion	−0.07 ± (0.06)	−0.07 ± (0.09)	= 0.922
		Plantarflexion	−1.41 ± (0.32)	−1.38 ± (0.32)	= 0.677
	Velocity (◦/s)	Dorsiflexion	109.21 ± (41.46)	105.07 ± (39.36)	= 0.632
		Plantarflexion	−154.38 ± (92.27)	−161.85 ± (83.24)	= 0.693
Coronal planes	Angle (◦)	Eversion	−10.97 ± 1.89	−13.05 ± 3.55	<0.001*
		Inversion	2.02 ± 6.28	−0.82 ± 6.65	0.25
	Moment (Nm/kg)	Eversion	0.02 ± 0.05	−0.01 ± 0.12	0.100
		Inversion	0.40 ± 0.17	0.45 ± 0.27	0.343
	Velocity (◦/s)	Eversion	−170.49 ± 46.36	−135.36 ± 51.84	0.53
		Inversion	291.09 ± 79.68	220.58 ± 54.26	<0.001*

“*” indicates a significant difference between FT, and MT, in the stance phase (*p* < 0.05).

### 3.2 Knee angle

The SPM analysis revealed in the knee angle that there was no significant difference between FT and ET in both the sagittal and coronal planes during the stance phase.

### 3.3 Muscle force

The SPM analysis revealed the results of muscle force during the stance phase, comparing FT dancers with ET dancers. Tibialis Posterior: (44.84%–50.23%, *p* = 0.016); Lateral Gastrocnemius: (4.08%–9.17%, *p* = 0.020) (43.25%–51.32%, *p* = 0.005); Medial Gastrocnemius (1.60%–10.15%, *p* = 0.006) (40.89%–48.70%, *p* = 0.008); SPM analysis revealed in the Tibialis Anterior, Peroneus Longus, Peroneus Brevis that there was no significant difference between FT and ET during the stance phase. Table 2 displays significant differences in the Tibialis Posterior between FT dancers and ET dancers (*p* < 0.027).

**TABLE 2:** Comparison of all muscle force variables change between FT dancers and ET dancers during the stance phase.

Peak muscle force (BW)	FT mean ± SD	ET mean ± SD	*p*-value
Tibialis Anterior	3.36 ± 1.07	3.17 ± 1.04	= 0.434
Tibialis Posterior	1.71 ± 0.43	1.91 ± 0.38	<0.027*
Peroneus Longus	1.66 ± 0.35	1.60 ± 0.48	= 0.537
Peroneus Brevis	0.82 ± 0.13	0.77 ± 0.11	= 0.138
Lateral Gastrocnemius	0.95 ± 0.16	1.02 ± 0.14	= 0.116
Medial Gastrocnemius	2.07 ± 0.46	1.96 ± 0.27	= 0.288

“*” indicates a significant difference between FT, and ET, in the stance phase (*p* < 0.05); Body Weight: BW.

### 3.4 Muscle co-activation ratio

Figure 6 shows the difference in the muscle co–activation ratio (%) between FT dancers and ET dancers during the descending phase, and the SPM analysis revealed that FT depicted a significantly smaller co-activation ratio than ET around ankle during the descending phase.

### 3.5 Center of mass (COM)

In the horizontal: Figure 7 shows the difference in the COM vertical position and velocity between FT dancers and ET dancers during the stance phase. The SPM analysis revealed that FT exhibited postural performance with a significantly higher CoM at almost 60% of the stance compared to ET, while there was no significant difference in velocity during the stance phase. In addition, Figure 8 shows that there was no significant difference in COM displacement between FT (13.60 ± 0.70 cm) and ET (13.20 ± 0.30 cm) during the stance phase.

## 4 Discussion

This study has investigated the biomechanical differences in the lower limb stance patterns between FT (forefoot) dancers and ET (entire foot) dancers. Initially, our hypothesis suggested that FT dancers might face a heightened susceptibility to ankle sprains in comparison to ET dancers. Our findings substantiated this hypothesis, revealing that FT dancers displayed a more pronounced plantarflexion angle, inversion angle, and inversion velocity in their ankle joint in contrast to ET dancers. Moreover, the FT dancers exhibited reduced co-activation of the muscles surrounding the ankle joint compared to their ET counterparts. These outcomes corroborate our initial assumptions and contribute substantially to comprehending the mechanisms underlying the risk of lower limb injuries among these distinct types of dancers from a biomechanical standpoint.

Previous studies have demonstrated a direct correlation, establishing that increased heel height corresponds with amplified plantarflexion activity within the ankle joint (Polat and Kabakcı, 2021). When FT dancers adopt the forefoot landing method, they effectively extend the distance between their heels and the ground, akin to the effect of wearing higher heels. As a result, this technique induces an escalated degree of ankle joint plantarflexion. Our study findings concur with this observation, notably illustrating that FT dancers consistently displayed a larger plantarflexion angle compared to ET dancers throughout the stance phase (*p* < 0.001). We speculate that the diminished dorsiflexion angle observed in the ankle joint of FT dancers might be attributed to two factors. Firstly, dancers limit the ankle joint’s range of motion to sustain bodily balance during high-speed movements. Secondly, the partial forefoot contact method used by dancers decreases the contact area between the ankle joint and the ground, enabling swifter directional changes and body movement transitions. Past studies have indicated that a reduced ankle dorsiflexion angle corresponds with increased peak landing forces, thus elevating stress around the ankle joint (Fong et al., 2011). Over an extended dance career, subjecting the ankle joint to prolonged periods of high-pressure conditions may result in fatigue, potentially heightening susceptibility to ankle sprains. In addition, another reason for the reduced dorsiflexion angle in FT dancers may be attributed to the influence of the long loop reflex. The decrease in contact area with the ground may impact stability reflexes triggered by somatosensory stimuli, resulting in a sensory loss experienced in the fusion of an upright stance (Binder et al., 2009). To maintain body stability, the long loop reflex engages in motor control and posture regulation by reducing the range of motion in the ankle joint to preserve balance.

Various modes of stance can exert an influence on the body’s stability (Horak et al., 1990; Winter et al., 1996), with the Center of Mass (COM) standing out as a pivotal metric for the assessment of human stability (Willaert et al., 2024). During the execution of kicking movements, the most profound alterations in the COM manifest in the vertical dimension. Rigorous computations of the vertical COM displacement, revealing no marked disparities in the transformations between FT and ET dancers (as shown in Figure 1). This phenomenon may be attributed to the similarity of Latin dance kicking motions to stationary single-leg ankle movements. With singular limb support, the muscle groups responsible for equilibrium maintenance exhibit heightened activity (Alfuth and Gomoll, 2018), inducing a subtle COM shift towards the supporting limb. The body endeavors to uphold equilibrium through nuanced muscle adjustments (Kaye and Jahss, 1991; Murley et al., 2014), implicating the neural system’s governance of the body in the vertical plane. While these movements contribute to the conditioning of lower limb musculature and the refinement of balance, the COM displacement is apt to be inconspicuous due to the singular support point. Under usual circumstances, such variations are typically governable and improbable to give rise to perceptible complications. Hence, our primary emphasis is directed towards discerning alterations in muscular activity.

The stance area of the lower limbs upon landing is noticeably smaller in FT dancers compared to ET dancers, potentially resulting in alterations in the musculoskeletal structure of the lower limbs (Polat and Kabakcı, 2021). These changes can significantly affect muscle force distribution around the ankle joint, to the extent of affecting the dancer’s balance. Our study found no significant differences between FT and ET dancers in the tibialis anterior muscle. However, during the (44.84%–50.23%, *p* = 0.016) stance phase, we noted slightly lower muscle force in the tibialis posterior muscle among FT dancers compared to ET dancers. The tibialis posterior muscle, situated in the lower leg’s posterior part, extends to the inner aspect of the foot, contributing to maintaining the foot arch’s concave shape and providing enhanced stance, balance, and load dispersion (Kaye and Jahss, 1991; Murley et al., 2014). Reduced muscle strength may result in inadequate control of ankle joint inversion angles during the stance phase, subsequently leading to increased inversion velocity (Murley et al., 2014). Our findings revealed that FT dancers demonstrated greater inversion angles than ET dancers during the (0%–9.83%, *p* = 0.001), (44.34%–79.52%, *p* = 0.003), and (88.56%–100%, *p* = 0.037) stance phases. Additionally, FT dancers exhibited higher inversion velocities, reaching a peak velocity of 291.09°/s during the initial ground contact phase. This increased inversion angle upon initial ground contact in FT dancers, possibly due to insufficient muscle strength, leads to challenges in controlling inversion speed, thereby elevating the risk of ankle injuries upon landing (Lin et al., 2019). Previous reports have highlighted that greater inversion angles and velocities are primary factors contributing to ankle joint sprains (Xu et al., 2022). Consequently, FT dancers may face an increased risk of ankle joint injuries (Gehring et al., 2014). The plantar fascia is a type of connective tissue located on the sole of the foot, playing a crucial role in controlling the complex tension of the metatarsal arch through the windlass mechanism (Hicks, 1954). It is pivotal in maintaining the shape of the foot and providing support by supporting and sustaining the concave structure of the foot arch through the intricate movements of the windlass mechanism. Additionally, the plantar fascia is involved in absorbing and transmitting body weight during walking and movement, while simultaneously maintaining the stability of the foot (Menz et al., 2005; Kleiner et al., 2011). The uneven distribution of foot strength in FT dancers may increase the load on the plantar fascia, leading to inflammation or strain. Therefore, this could also be one of the factors influencing the balance of FT dancers. In the future, we can delve deeper into the physiological mechanisms and related mechanics of Latin dancers’ feet, especially those related to ankle joint stability.

Our study found notable differences in muscle force between FT and ET dancers. Specifically, FT dancers exhibited lower lateral gastrocnemius muscle force than ET dancers during the (4.08%–9.17%, *p* = 0.020) and (43.25%–51.32%, *p* = 0.005) stance phases. Similarly, reduced medial gastrocnemius muscle force was observed in FT dancers compared to ET dancers during the (1.60%–10.15%, *p* = 0.006) and (40.89%–48.70%, *p* = 0.008) stance phases. The gastrocnemius muscle plays a crucial role in mitigating excessive lateral ankle deviation in response to external forces or ankle eversion tendencies, thereby reducing susceptibility to ankle inversion sprains (Lin et al., 2019). This mechanism is particularly important in preventing ankle injuries within the realm of Latin dance. The intricate footwork involved in Latin dance routines, encompassing external and internal rotations, swift directional changes, and rotational maneuvers, places a substantial demand on ankle joint stability. Inadequate strength in the peroneal muscles to counteract external forces inherent in these complex movements significantly increases susceptibility to ankle sprains.

In this study, we utilized electromyography (EMG) to validate the OpenSim model, revealing a strong correlation between predicted muscle activations by the model and the actual EMG recordings. This alignment allowed us to identify both agonist and antagonist muscles involved in the movements under study. Leveraging muscle activation data extracted from OpenSim, we applied established methodologies to calculate muscle co-activation in both FT and ET dancers (Lin et al., 2019). Our analysis primarily focused on muscles surrounding the knee and ankle joints, revealing lower co-activation around the ankle, notably in SOL/TA and MG/TA, among FT dancers. This signifies less ankle joint stability and poorer muscle control in this group. Studies have highlighted that lower limb joint stability heavily relies on muscle co-activation (Pan et al., 2023; Yang et al., 2023). Particularly crucial for Latin dancers, swift weight shifts between feet are common in this dance style, such as during kicks performed in the jive. These movements demand rapid transitions between ankle dorsiflexion and plantarflexion. Hence, maintaining robust stability is fundamental for providing adequate support and forms the bedrock of a dancer’s movement quality. The discerned subjective preference upon landing in FT mode, particularly the discernible reduction in moment arms along the longitudinal and anteroposterior axes, may be intricately linked to individuals’ perceptual acuity and adaptive prowess in the realm of posture control. The extant literature posits that as the support base diminishes, a paradigmatic shift occurs in the dominant mode of posture control, transitioning from the ankle joint towards more proximal anatomical structures, notably adopting knee or hip-centric strategies (Keshner and Allum, 1990). Within the purview of our investigation, we observed a lack of statistically significant alterations in knee joint angles during FT mode. Nonetheless, a conspicuous augmentation in the co-activation of muscles encircling the knee joint was noted, indicative of an adaptive response to the distinctive support paradigm inherent in FT. This phenomenon potentially serves as a pivotal determinant for dancers navigating FT mode to uphold stability. Consequently, despite the absence of overt changes in knee joint angles, adaptation manifested at the level of muscular co-activation, thereby contributing substantively to stability maintenance in FT mode and proffering a plausible elucidation for the attested subjective preference. This discovery underscores the pivotal role of muscular activity and co-activation within the milieu of posture control research, especially in the context of adaptive modulations when confronted with divergent stance patterns.

While the current study extensively explored various aspects of Latin dance biomechanics, including the intricate footwork and its impact on ankle joints, the trajectories of the free functional leg (left) in terms of movement variability were not specifically analyzed in this research. This indeed represents a limitation of our study. Examining the trajectories of the free leg could potentially provide valuable insights into the preferred stance modality of individual participants, offering a more comprehensive understanding of their movement patterns. We acknowledge this limitation and consider it an avenue for future research to delve deeper into the nuanced aspects of Latin dance biomechanics.

## 5 Conclusion

This study biomechanically demonstrates that in various stance patterns within Latin dance, a reduction in lower limb stance area leads to weakened muscle strength and reduced co-activation around the ankle joint, and results in increased ankle inversion angles and velocities, thereby heightening the risk of ankle sprains. The diminished muscle strength and co-activation affect their ability to effectively control the inward and outward movements of the ankle, resulting in reduced regulation of ankle joint motion. The increased co-activation around the knee joint in FT dancers may be a compensatory response for reducing the lower limb stance area in order to maintain stability. As a result, FT dancers may significantly heighten their risk of ankle sprains during dance activities. To mitigate such risks, dancers should prioritize safeguarding the health of their ankle joints. This involves not only mastering proper dance techniques and postures but also integrating customized strength training specifically targeting ankle joint stability. This proactive approach is crucial for maintaining balance and injury resistance during rigorous dance training and performances.

## Data Availability

The original contributions presented in the study are included in the article/Supplementary material, further inquiries can be directed to the corresponding authors.
